# Association between diabetes status and subsequent onset of glaucoma in postmenopausal women

**DOI:** 10.1038/s41598-021-97740-3

**Published:** 2021-09-14

**Authors:** Younhea Jung, Kyungdo Han, Kyoung Ohn, Da Ran Kim, Jung II Moon

**Affiliations:** 1 Department of Ophthalmology, Yeouido St. Mary’s Hospital, College of Medicine, The Catholic University of Korea, 10, 63-ro, Yeongdeungpo-gu, Seoul, 07345 Republic of Korea; 2 Department of Statistics and Actuarial Science, Soongsil University, Seoul, Republic of Korea

**Keywords:** Optic nerve diseases, Diabetes

## Abstract

The purpose of this study was to analyze the risk of glaucoma based on diabetes status using a large nationwide longitudinal cohort of postmenopausal women. This study included 1,372,240 postmenopausal women aged ≥ 40 years who underwent National Health Screening Program in 2009. Subjects were classified into the following 5 categories based on diabetes status: no diabetes, impaired fasting glucose (IFG), new onset diabetes, diabetes treated with oral hypoglycemic medication, and diabetes treated with insulin. Subjects were followed from 2005 through 2018, and hazard ratios of glaucoma onset were calculated for each group. Subgroup analyses of subjects stratified by age, smoking, drinking, hypertension, and dyslipidemia were performed. During the follow up period, 42,058 subjects developed glaucoma. The adjusted hazard ratio was 1.061 (95% CI, 1.036–1.086) in the IFG group, 1.151 (95% CI, 1.086–1.220) in the new onset diabetes group, 1.449 (95% CI, 1.406–1.493) in the diabetes treated with oral hypoglycemic medication group, and 1.884(95% CI, 1.777–1.999) in the diabetes treated with insulin group compared to the no diabetes group. The results were consistent in subgroup analyses after stratifying by age, lifestyle factors (smoking and drinking), and comorbidities (hypertension and dyslipidemia). Diabetes status is associated with increased risk of glaucoma development in postmenopausal women.

## Introduction

Diabetes is a global health burden^1^. Although there are various treatments developed to control plasma glucose, the prevalence of diabetes and its macrovascular and microvascular complications is increasing worldwide^1,2^. Glaucoma is also a major burden, which is characterized by irreversible progressive visual field loss corresponding to the loss of retinal ganglion cells, and most patients are asymptomatic until late stage. Therefore, identifying its risk factors for early detection of glaucoma is clinically important^3^.

Diabetes has been suggested to increase the risk of glaucoma by increasing intraocular pressure (IOP)^4–6^. However, the association between diabetes and IOP was weak in previous studies, and furthermore, the most prevalent type of glaucoma in Korea is normal tension glaucoma^7–9^. In this regard, diabetes has also been suggested to cause microvascular damage and vascular dysregulation of the optic nerve head which increases the susceptibility to glaucomatous damage^4–6^.

Many prior studies report conflicting results on the association between diabetes and glaucoma^10–13^. In addition, there is a relative lack of studies associating glaucoma and impaired fasting glucose (IFG) or effect of treatment for diabetes such as oral hypoglycemic agents or insulin^8^.

Therefore, we conducted a longitudinal study to analyze the risk of glaucoma development according to diabetes status. Specifically, we used a large nationwide cohort of postmenopausal women to determine the risk of glaucoma onset in subjects with no diabetes, IFG, new onset diabetes, diabetes treated with oral hypoglycemic medication, or diabetes treated with insulin.

## Methods

### Data source

This study was based on the Korean National Health Insurance Service (KNHIS) database. The KNHIS is a single insurer managed by the Korean government, and provides comprehensive medical care to 97% of the Korean population^14^. The database contains demographics (anonymized code for each individual, age, sex, socioeconomics, household income, etc.) and medical data (inpatient and outpatient service records, diagnostic codes classified by the International Classification of Diseases 10^th^ revision [ICD-10], prescriptions, and medical procedures).

The KNHIS also provides a breast cancer check-up along with standardized National Health Screening Program (NHSP) to all women aged ≥ 40 years insured by the KNHIS. The program includes anthropometric data, a set of laboratory tests, and a self-reported questionnaire with regards to health behaviors. Additionally, it also includes a comprehensive survey encompassing clinical symptoms, weight loss, family history of cancer, and various reproductive factors (age at menarche, age at menopause, history of hormone replacement therapy, parity, breastfeeding, history of oral contraceptive use, etc.).

This study was approved by the Institutional Review Board of the Yeouido St. Mary’s Hospital, Seoul, Korea, which waived consent from individual subjects because we used publicly open and anonymized data. Our research adhered to the tenets of the Declaration of Helsinki.

### Study population

**Figure 1:**
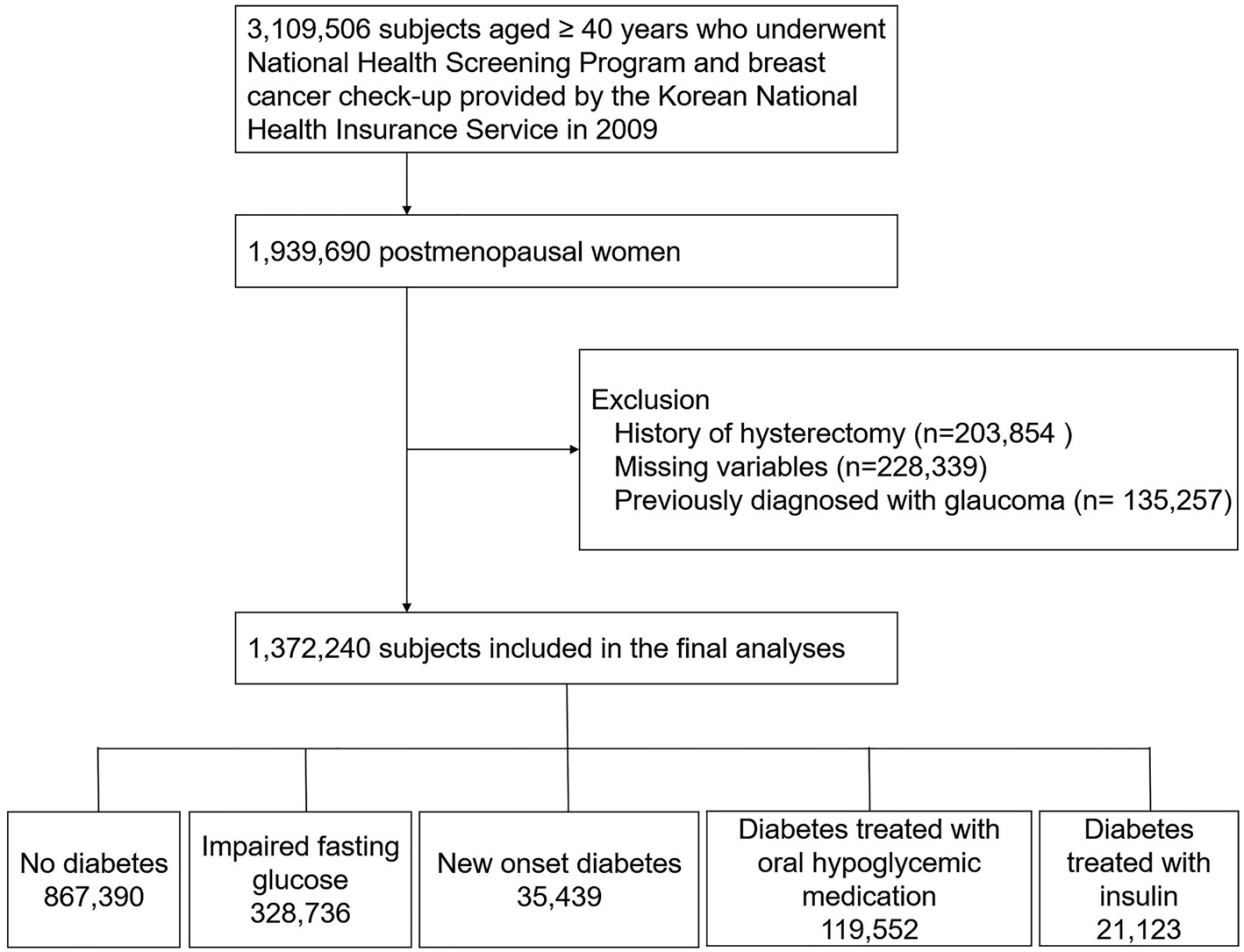
Study subjects.

In the study, 3,109,506 women aged ≥ 40 years who had undergone NHSP and breast cancer check-up in 2009 (index year) were initially screened (Fig. 1). We used this database, because many covariates were recorded at this check-up. Of these, we selected 1,939,690 subjects who were postmenopause. Subjects with unnatural menopause (n = 203,854) due to hysterectomy or those with missing variables (n = 228,339) were excluded. Subjects with previously diagnosed glaucoma before the index year (n = 135,257) were also excluded. A total of 1,372,240 naturally postmenopausal women without prior history of glaucoma were included in the final analyses and were followed until December 31, 2018. Subjects were censored if they developed glaucoma or died.

Type 2 diabetes was defined using a combination of KNHID claims data and NHSP results. History of diabetes diagnosis was defined as at least one claim per year with E11-E14 (ICD-10 codes) and at least one claim per year with prescription for antidiabetic medication (sulfonylureas, metformin, meglitinides, thiazolidinediones, dipeptidyl peptidase-4 inhibitors, α-glucosidase inhibitors, or insulin^15^. Diabetes status was stratified into 5 categories using the data within one year prior to the index date: (1) normal (no history of diabetes diagnosis and FBS < 100 mg/dL), (2) IFG (no history of diabetes diagnosis and 100 mg/dL ≤ FBS < 126 mg/dL), (3) new onset diabetes (no history of diabetes diagnosis and FBS ≥ 126 mg/dL), (4) diabetes treated with oral hypoglycemic medication, and (5) diabetes treated with insulin. If a subject was using oral hypoglycemic medication and insulin together, he/she was categorized into the insulin group.

The primary end point was development of glaucoma, which was defined based on ICD-10 code for primary open-angle glaucoma (H401). Those with at least 3 visits for glaucoma were included in the study to enhance the validity of the diagnosis^16^.

Covariates, which were measured during NHSP check-up in 2009, included age, smoking, drinking, exercise, body mass index, parity, breastfeeding, oral contraceptive use, age at menarche, age at menopause, and hormone replacement therapy.

Smoking status was classified into nonsmoker, ex-smoker, or current smoker using the following question: “Have you ever smoked more than five packs of cigarettes in your life?”^17^. Alcohol drinking was categorized as no alcohol, mild alcohol (< 30 g per day), or heavy alcohol (≥ 30 g per day). Low income was defined as an annual household income level in the lowest quintile. Regular exercise was defined as moderate-intensity exercise for ≥ 30 min, ≥ 5 times a week or vigorous-intensity exercise for ≥ 20 min, ≥ 3 times a week. Participants’ body mass index (BMI) was calculated as weight (kg) divided by the square of height (m^2^). Systolic and diastolic BP were measured in a seated position after resting more than 5 min. Fasting glucose and total cholesterol were measured with blood samples collected after an overnight fasting.

Comorbidities, including hypertension and dyslipidemia, were based on ICD-10 codes within one year prior to NHSP and NHSP results. Hypertension was defined based on ICD-10 code for hypertension (I10-I13 and I15) with at least one prescription for antihypertensive medication or systolic BP ≥ 140 mmHg or diastolic BP ≥ 90 mmHg. Dyslipidemia was defined as at least one prescription of lipid-lowering medication under ICD-10 code for dyslipidemia (E78) or as serum total cholesterol level ≥ 240 mg/dL.

### Statistical analysis

All statistical analyses were conducted using SAS (ver 9.4; SAS Institute, Cary, NC, USA) with *P* values < 0.05 considered significant.

The baseline characteristics of the study participants were compared in 5 diabetes groups using the Student *t*-test and ANOVA for continuous variables and χ^2^ test for categorical variables. The incidence rates of endpoint outcome were calculated by dividing the number of events by 1,000 person-years. Cox proportional hazard regression analyses were used to calculate the risk of glaucoma onset according to diabetes status. Hazard ratios (HR) and 95% confidence intervals (CI) of endpoint outcomes were derived before and after adjusting for potential confounding factors. Subgroup analyses were performed after stratifying by age, smoking, drinking, hypertension, and dyslipidemia. To test the significance of the subgroup effects, interaction terms of diabetes status with age, smoking, drinking, hypertension, and dyslipidemia status were added to the Cox model, respectively, and *P*-values for interaction were reported. Fully-adjusted model included age, income, smoking, drinking, exercise, body mass index, and reproductive factors including parity, breastfeeding, oral contraceptive use, age at menarche, age at menopause, and hormone replacement therapy. Kaplan–Meier curves for incidence probabilities of glaucoma according to diabetes status were generated.

## Results

### Baseline characteristics of the study population

A total of 1,372,240 subjects, who underwent natural menopause, were included in the study (Fig. 1). The baseline characteristics of the study population are shown in Table 1. Compared to subjects without diabetes, those with diabetes were older, more likely to be ex- or current smokers, and more obese.

**Table 1:** Baseline characteristics.

*N*	Diabetes status				
	No diabetes	Impaired fasting glucose	New onset diabetes	Diabetes treated with oral hypoglycemic medication	Diabetes treated with insulin
	867,390	328,736	35,439	119,552	21,123
**Demographics**					
Age	60.66 ± 8.20	61.75 ± 8.25	62.83 ± 8.52	64.80 ± 7.75	65.24 ± 7.75
Smoking					
Non	835,933 (96.37)	315,689 (96.03)	33,759 (95.26)	114,637 (95.89)	20,200 (95.63)
Ex	8890 (1.02)	3697 (1.12)	390 (1.10)	1431 (1.20)	276 (1.31)
Current	22,567 (2.60)	9350 (2.84)	1290 (3.64)	3484 (2.91)	647 (3.06)
Alcohol drinking					
No	757,370 (87.32)	281,904 (85.75)	30,436 (85.88)	109,844 (91.88)	19,899 (94.21)
Mild	106,102 (12.23)	44,430 (13.52)	4690 (13.23)	9264 (7.75)	1178 (5.58)
Heavy	3918 (0.45)	2402 (0.73)	313 (0.88)	444 (0.37)	46 (0.22)
Regular Exercise	161,459 (18.61)	59,471 (18.09)	6053 (17.08)	22,648 (18.94)	3705 (17.54)
Income (lowest quintile)	200,051 (23.06)	74,451 (22.65)	8369 (23.62)	25,618 (21.43)	4258 (20.16)
**Past medical history**					
Diabetes mellitus	0 (0)	0 (0)	35,439 (100)	119,552 (100)	21,123 (100)
Hypertension	336,269 (38.77)	167,296 (50.89)	21,234 (59.92)	85,631 (71.63)	16,141 (76.41)
Dyslipidemia	248,890 (28.69)	126,491 (38.48)	15,711 (44.33)	64,910 (54.29)	12,662 (59.94)
Chronic kidney disease	85,653 (9.87)	41,059 (12.49)	5373 (15.16)	21,144 (17.69)	5677 (26.88)
**Laboratory findings**					
Systolic blood pressure, mmHg	123.93 ± 15.90	127.62 ± 16.12	130.28 ± 16.73	129.77 ± 16.15	129.52 ± 16.85
Diastolic blood pressure, mmHg	76.21 ± 10.10	78.04 ± 10.18	79.16 ± 10.38	77.87 ± 10.04	77.02 ± 10.37
Fasting blood glucose, mg/dL	88.62 ± 7.25	107.84 ± 6.56	147.83 ± 33.30	133.88 ± 42.33	148.93 ± 63.20
Cholesterol, mg/dL	206.98 ± 41.99	214.58 ± 45.38	218.92 ± 48.19	199.36 ± 48.42	195.48 ± 52.1
Body mass index, kg/cm^2^	23.8 ± 2.99	24.52 ± 3.15	25.08 ± 3.36	25.19 ± 3.35	24.86 ± 3.42
**Reproductive factors**					
Age at menarche, y	16.41 ± 1.84	16.48 ± 1.83	16.56 ± 1.82	16.58 ± 1.81	16.59 ± 1.80
− 11	8746 (1.01)	3037 (0.92)	286 (0.81)	917 (0.77)	160 (0.76)
12–15	452,384 (52.15)	166,555 (50.67)	17,314 (48.86)	57,589 (48.17)	10,007 (47.37)
16-	406,260 (46.84)	159,144 (48.41)	17,839 (50.34)	61,046 (51.06)	10,956 (51.87)
Age at menopause, y	49.98 ± 3.95	50.08 ± 4.02	50.06 ± 4.17	50.1 ± 4.32	49.9 ± 4.46
− 39	14,430 (1.66)	5352 (1.63)	675 (1.90)	2525 (2.11)	515 (2.44)
40–44	48,832 (5.63)	18,581 (5.65)	2148 (6.06)	7465 (6.24)	1472 (6.97)
45–49	239,985 (27.67)	88,770 (27.00)	9195 (25.95)	30,284 (25.33)	5466 (25.88)
50–54	477,880 (55.09)	179,398 (54.57)	19,271 (54.38)	63,299 (52.95)	10,918 (51.69)
55-	86,263 (9.95)	36,635 (11.14)	4150 (11.71)	15,979 (13.37)	2752 (13.03)
Total reproductive years	33.57 ± 4.33	33.6 ± 4.41	33.5 ± 4.57	33.52 ± 4.72	33.31 ± 4.83
− 29	116,954 (13.48)	45,177 (13.74)	5161 (14.56)	18,086 (15.13)	3458 (16.37)
30–34	364,967 (42.08)	136,751 (41.60)	14,731 (41.57)	48,688 (40.73)	8796 (41.64)
35–39	333,568 (38.46)	124,948 (38.01)	13,070 (36.88)	43,284 (36.21)	7224 (34.20)
40-	51,901 (5.98)	21,860 (6.65)	2477 (6.99)	9494 (7.94)	1645 (7.79)
Hormone replacement therapy					
None	691,411 (79.71)	268,870 (81.79)	29,991 (84.63)	101,322 (84.75)	17,805 (84.29)
< 2 years	83,868 (9.67)	28,849 (8.78)	2501 (7.06)	8101 (6.78)	1399 (6.62)
2–5 years	35,389 (4.08)	11,262 (3.43)	961 (2.71)	3063 (2.56)	543 (2.57)
≥ 5 years	27,363 (3.15)	7929 (2.41)	692 (1.95)	2666 (2.23)	501 (2.37)
Unknown	29,359 (3.38)	11,826 (3.60)	1294 (3.65)	4400 (3.68)	875 (4.14)
Parity					
None	22,117 (2.55)	8122 (2.47)	817 (2.31)	2431 (2.03)	410 (1.94)
1	55,875 (6.44)	19,628 (5.97)	1969 (5.56)	5080 (4.25)	899 (4.26)
≥ 2	789,398 (91.01)	300,986 (91.56)	32,653 (92.14)	112,041 (93.72)	19,814 (93.80)
Breastfeeding					
None	60,027 (6.92)	21,537 (6.55)	2140 (6.04)	6013 (5.03)	1045 (4.95)
< 6 months	61,690 (7.11)	20,906 (6.36)	1830 (5.16)	5011 (4.19)	833 (3.94)
6 months-1 year	157,643 (18.17)	56,712 (17.25)	5560 (15.69)	16,664 (13.94)	2934 (13.89)
≥ 1 year	588,030 (67.79)	229,581 (69.84)	25,909 (73.11)	91,864 (76.84)	16,311 (77.22)
Oral contraceptive use					
None	696,490 (80.30)	261,485 (79.54)	28,383 (80.09)	93,811 (78.47)	16,506 (78.14)
< 1 year	78,350 (9.03)	30,324 (9.22)	3051 (8.61)	10,811 (9.04)	1831 (8.67)
≥ 1 year	50,104 (5.78)	20,468 (6.23)	2205 (6.22)	8828 (7.38)	1612 (7.63)
Unknown	42,446 (4.89)	16,459 (5.01)	1800 (5.08)	6102 (5.10)	1174 (5.56)

All baseline characteristics are statistically significant at 0.001.

**Figure 2:**
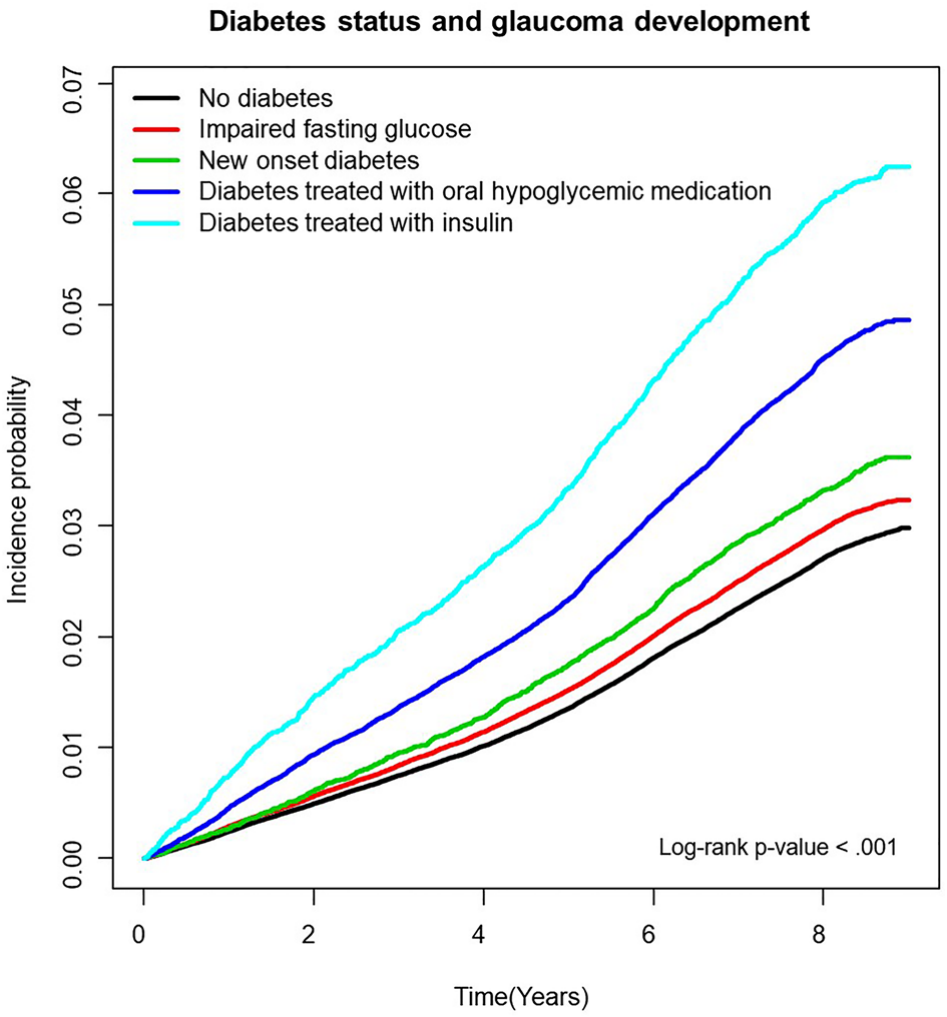
Cumulative incidence of glaucoma according to diabetes status.

Table 2 shows the risk of glaucoma in postmenopausal women according to diabetes status before and after adjusting for confounding factors. The unadjusted risk of incident glaucoma increased according to diabetes status: in IFG (hazard ratio [HR] = 1.097, 95% confidence interval [CI] = 1.072–1.123), in new onset diabetes (HR = 1.226, 95% CI = 1.157–1.300), diabetes treated with oral hypoglycemic medication (HR = 1.672, 95% CI = 1.624–1.722), diabetes treated with insulin (HR = 2.200, 95% CI = 2.075–2.333) in non-adjusted model. After adjusting for confounding factors (age, income, smoking, drinking, exercise, body mass index, and reproductive factors including parity, breastfeeding, oral contraceptive use, age at menarche, age at menopause, and hormone replacement therapy), the adjusted HR of glaucoma was 1.061 (95% CI = 1.036–1.086), 1.151 (95% CI = 1.086–1.220), 1.449 (95% CI = 1.406–1.493), and 1.884 (95% CI = 1.777–1.999) in IFG, new onset diabetes, diabetes treated with oral hypoglycemic medication, and diabetes treated with insulin, respectively. The cumulative incidence of glaucoma according to diabetes status is shown in Fig. 2.

**Table 2:** Risk of glaucoma according to diabetes status.

Diabetes status	N	Glaucoma	Duration	Incidence rate*	Model 1	Model 2	Model 3
No diabetes	867,390	24,264	7,098,664.23	3.418	1 (Ref.)	1 (Ref.)	1 (Ref.)
Impaired fasting glucose	328,736	10,023	2,675,849.58	3.745	1.097 (1.072,1.123)	1.058 (1.034,1.083)	1.061 (1.036,1.086)
New onset diabetes	35,439	1187	283,958.39	4.180	1.226 (1.157,1.300)	1.141 (1.076,1.210)	1.151 (1.086,1.220)
Diabetes treated with oral hypoglycemic medication	119,552	5410	951,438.58	5.686	1.672 (1.624,1.722)	1.445 (1.402,1.489)	1.449 (1.406,1.493)
Diabetes treated with insulin	21,123	1174	158,184.39	7.421	2.2 (2.075,2.333)	1.882 (1.774,1.996)	1.884 (1.777,1.999)
*P*-value					< .0001	< .0001	< .0001
*	per 1000						
Model 1	Non-adjusted						
Model 2	Adjusted for age, income, smoking, drinking, exercise, body mass index						
Model 3	Adjusted for age, income, smoking, drinking, exercise, body mass index, and reproductive factors including parity, breastfeeding, oral contraceptive use, age at menarche, age at menopause, and hormone replacement therapy

**Figure 3:**
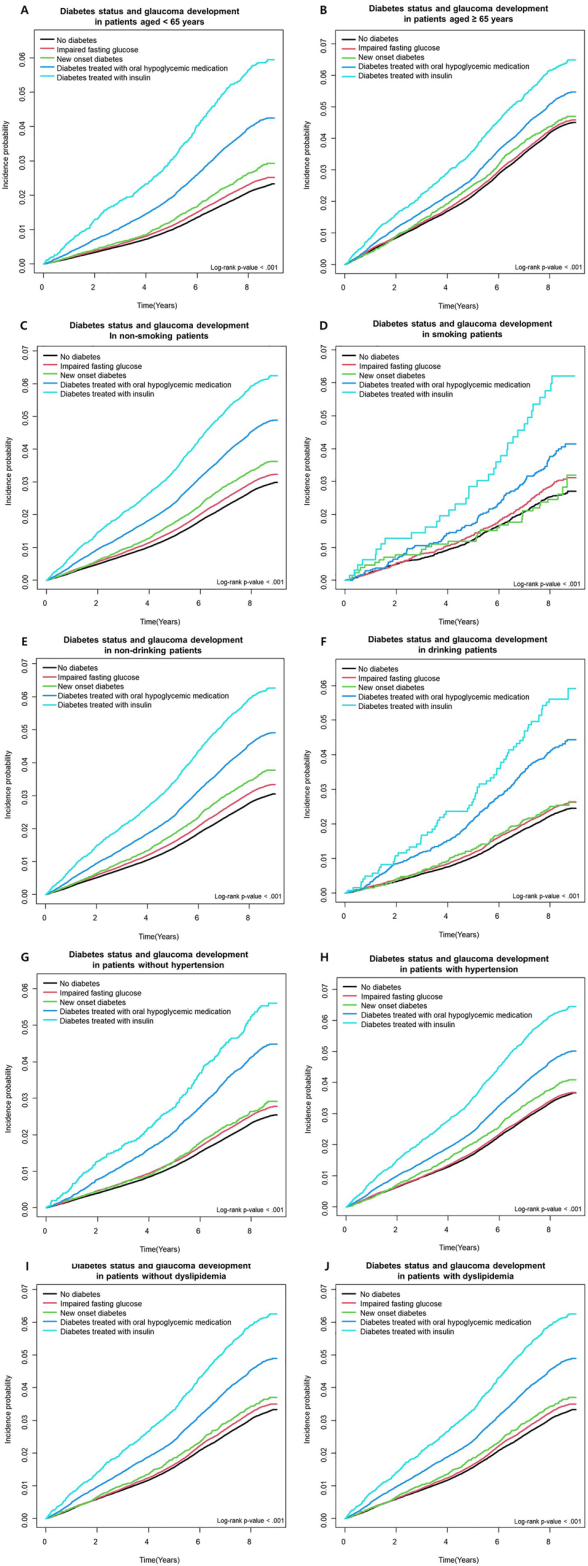
Cumulative incidence of glaucoma according to diabetes status in subgroups after stratifying by age (**A** and **B**), smoking (**C** and **D**), drinking (**E** and **F**), hypertension (**G** and **H**), and dyslipidemia (**I** and **J**).

The comparison of adjusted HRs (96% CIs) of glaucoma incidence in subgroups after stratifying by age, smoking, drinking, hypertension, and dyslipidemia is shown in Table 3. The association between diabetes status and subsequent glaucoma was consistent in all subgroup analyses. The risk of incident glaucoma was more prominent in younger age group (*P* for interaction < 0.001) compared to older age group and those without hypertension (*P* for interaction < 0.001) compared to those with hypertension. Figure 3 shows the cumulative incidence of glaucoma according to diabetes status in subgroups.

**Table 3:** Risk of glaucoma according to diabetes status stratified by age, smoking, drinking, hypertension, and dyslipidemia.

	Diabetes status	N	Glaucoma	Duration	Incidence rate*	Model	*P* for interaction
**Age**							
Age < 65	No diabetes	602,698	13,225	4,990,467.84	2.650	1 (Ref.)	< .001
	Impaired fasting glucose	213,597	5152	1,764,649.11	2.920	1.076 (1.042,1.111)	
	New onset diabetes	21,325	586	174,819.21	3.352	1.228 (1.130,1.334)	
	Diabetes treated with oral hypoglycemic medication	58,817	2401	481,406.54	4.987	1.666 (1.594,1.741)	
	Diabetes treated with insulin	9705	544	76,659.99	7.096	2.357 (2.162,2.569)	
Age ≥ 65	No diabetes	264,692	11,039	2,108,196.38	5.236	1 (Ref.)	
	Impaired fasting glucose	115,139	4871	911,200.47	5.346	1.032 (0.998,1.068)	
	New onset diabetes	14,114	601	109,139.19	5.507	1.085 (0.999,1.178)	
	Diabetes treated with oral hypoglycemic medication	60,735	3009	470,032.05	6.402	1.246 (1.197,1.298)	
	Diabetes treated with insulin	11,418	630	81,524.40	7.728	1.503 (1.387,1.628)	
**Smoking**							
No	No diabetes	844,823	23,700	6,917,972.06	3.426	1 (Ref.)	0.541
	Impaired fasting glucose	319,386	9754	2,601,311.44	3.750	1.058 (1.034,1.084)	
	New onset diabetes	34,149	1154	273,859.12	4.214	1.154 (1.087,1.224)	
	Diabetes treated with oral hypoglycemic medication	116,068	5282	924,409.81	5.714	1.450 (1.407,1.495)	
	Diabetes treated with insulin	20,476	1140	153,556.71	7.424	1.876 (1.767,1.991)	
Current	No diabetes	22,567	564	180,692.17	3.121	1 (Ref.)	
	Impaired fasting glucose	9350	269	74,538.15	3.609	1.163 (1.004,1.346)	
	New onset diabetes	1290	33	10,099.27	3.268	1.051 (0.739,1.494)	
	Diabetes treated with oral hypoglycemic medication	3484	128	27,028.77	4.736	1.369 (1.125,1.666)	
	Diabetes treated with insulin	647	34	4627.68	7.347	2.171 (1.533,3.075)	
**Drinking**							
No	No diabetes	757,370	21,707	6,191,490.16	3.506	1 (Ref.)	0.178
	Impaired fasting glucose	281,904	8856	2,291,470.84	3.865	1.063 (1.037,1.090)	
	New onset diabetes	30,436	1063	243,485.39	4.366	1.169 (1.099,1.244)	
	Diabetes treated with oral hypoglycemic medication	109,844	5005	872,936.23	5.734	1.443 (1.398,1.489)	
	Diabetes treated with insulin	19,899	1109	148,700.68	7.458	1.879 (1.768,1.996)	
Yes	No diabetes	110,020	2557	907,174.07	2.819	1 (Ref.)	
	Impaired fasting glucose	46,832	1167	384,378.74	3.036	1.042 (0.972,1.117)	
	New onset diabetes	5003	124	40,473.00	3.064	1.015 (0.847,1.216)	
	Diabetes treated with oral hypoglycemic medication	9708	405	78,502.35	5.159	1.544 (1.388,1.718)	
	Diabetes treated with insulin	1224	65	9483.71	6.854	2.020 (1.578,2.586)	
**Hypertension**							
No	No diabetes	531,121	12,784	4,375,071.01	2.922	1 (Ref.)	< .001
	Impaired fasting glucose	161,440	4233	1,324,433.19	3.196	1.075 (1.038,1.113)	
	New onset diabetes	14,205	383	115,159.74	3.326	1.078 (0.973,1.193)	
	Diabetes treated with oral hypoglycemic medication	33,921	1436	274,310.38	5.235	1.552 (1.469,1.640)	
	Diabetes treated with insulin	4982	256	38,665.23	6.621	1.951 (1.724,2.208)	
Yes	No diabetes	336,269	11,480	2,723,593.22	4.215	1 (Ref.)	
	Impaired fasting glucose	167,296	5790	1,351,416.39	4.284	1.020 (0.988,1.052)	
	New onset diabetes	21,234	804	168,798.65	4.763	1.139 (1.061,1.224)	
	Diabetes treated with oral hypoglycemic medication	85,631	3974	677,128.21	5.869	1.346 (1.298,1.396)	
	Diabetes treated with insulin	16,141	918	119,519.16	7.681	1.761 (1.646,1.884)	
**Dyslipidemia**							
No	No diabetes	618,500	16,446	5,063,804.87	3.248	1 (Ref.)	0.076
	Impaired fasting glucose	202,245	5821	1,645,528.60	3.537	1.053 (1.022,1.085)	
	New onset diabetes	19,728	643	157,626.60	4.079	1.163 (1.075,1.259)	
	Diabetes treated with oral hypoglycemic medication	54,642	2436	432,544.56	5.632	1.464 (1.402,1.529)	
	Diabetes treated with insulin	8461	463	62,460.74	7.413	1.930 (1.759,2.117)	
Yes	No diabetes	248,890	7818	2,034,859.36	3.842	1 (Ref.)	
	Impaired fasting glucose	126,491	4202	1,030,320.98	4.078	1.044 (1.006,1.084)	
	New onset diabetes	15,711	544	126,331.79	4.306	1.096 (1.005,1.196)	
	Diabetes treated with oral hypoglycemic medication	64,910	2974	518,894.02	5.731	1.368 (1.310,1.428)	
	Diabetes treated with insulin	12,662	711	95,723.65	7.428	1.751 (1.621,1.891)	
*	per 1000						
Model	Adjusted for age, income, smoking, drinking, exercise, body mass index, and reproductive factors including parity, breastfeeding, oral contraceptive use, age at menarche, age at menopause, and hormone replacement therapy

## Discussion

In this nationwide longitudinal cohort study of Korean adults with natural menopause, type 2 diabetes was associated with an increased risk of glaucoma incidence before and after adjusting for confounding factors. More importantly, diabetes status, stratified into IFG, new onset diabetes, diabetes treated with oral hypoglycemic medication, and diabetes treated with insulin, successively increased the risk of glaucoma. While these findings were consistent in all subgroups, the association was more prominent in younger age group (< 65 years) compared to older age group and those without hypertension compared to those with hypertension.

While there are previous studies on the association between diabetes and glaucoma or increased level of intraocular pressure, there is a relative lack of studies associating IFG and glaucoma, and the results are controversial^8^. Choi et al.^18^ reported increased incidence of glaucoma in those with high fasting glucose level. However, high glucose level ≥ 200 mg/dL was not associated with glaucoma in the Los Angeles Latino Eye Study^19^. Different measurement methods and different ethnicities may have resulted in various results across studies. Our study provides further evidence supporting that IFG also increases the risk of glaucoma development in naturally post-menopausal Korean women. However, the aHR was close to 1, namely 1.061, indicating about 6% increase in the risk of glaucoma development in patients with IFG compared to normal controls. It is worth noting that IFG was based on a single measurement of fasting blood glucose, and even small increase in HR, we believe, is clinically important. Our results suggest that incidence of glaucoma is proportional to the severity of glycemic burden, and this is the first study to report the association between IFG and glaucoma development in a national scale, longitudinal data.

The mechanisms associating diabetes or IFG to glaucoma are unclear. Hyperglycemia may increase IOP by drawing excess aqueous humor into the anterior chamber^20,21^, or by altering the trabecular meshwork function^6^. However, the association between diabetes and IOP in previous studies was weak^7,8^, and more importantly, in Korea, the most prevalent type of glaucoma is normal tension glaucoma, which suggest that there are other mechanisms beyond increased IOP. Vascular mechanisms also have been proposed to play a role between diabetes and glaucoma even in prediabetic stages^22^. It has been suggested that diabetes causes microvascular damage and vascular dysregulation of the optic nerve head and the retina, thereby increasing the susceptibility to glaucomatous damage^19,23^.

In addition, we found that diabetic patients treated with insulin (aHR = 1.884) were associated with higher risk of glaucoma compared to those treated with oral hypoglycemic agents (aHR = 1.449). This association was consistent even in all subgroup analyses before and after adjusting for confounding factors. Previous studies have also reported higher risk for glaucoma in diabetic patients treated with insulin^24–26^. Graw et al.^25^ reported an increased risk of glaucoma (odds ratio of 5.8) in diabetic patients treated with insulin and oral antidiabetics. The Thessaloniki Eye Study also revealed increased risk of glaucoma in those with history of diabetes treated with insulin compared to those treated without insulin ^24^. The Baltimore Eyes Study reported increased mean IOP in patients using insulin compared to those without diabetes. In previous studies, diabetes treated with insulin was a self-reported parameter which is subject to potential recall bias, however, in our study, treatment with insulin was based on objective criteria, ICD-10 codes. Although the mechanism is unclear, it could be an effect from insulin itself, or insulin may be a marker for diabetes severity indicating insulin resistance and high glycemic burden^24^. Our findings warrant further research.

While this robust association between diabetes and glaucoma was consistent in all subgroups classified by age, smoking or drinking status, hypertension or dyslipidemia, the association was more prominent in younger age group (< 65 years) compared to older age group and those without hypertension compared to those with hypertension.

This study is based on a large population-based epidemiologic cohort with a long follow up period. Our study design using long observation period and eliminating previously diagnosed glaucoma patients enabled us to explore the causality of diabetes status and glaucoma. However, there are also limitations. First, due to the use of claims and health examination database, we did not have access to clinical data regarding severity of diabetes or glaucoma such as HbA1c, IOP measurements, or visual field examinations. Second, our results may partly be affected by selection bias, indicating that diabetic patients may be more likely to receive more frequent eye examinations resulting in overestimation of the relationship between diabetes and glaucoma. In addition, our findings are based on only the Korean population where about 77% of primary open-angle glaucoma patients have normal IOP^9^ and we only included women. Therefore, our findings cannot be extrapolated to men or other populations where glaucoma results from increased IOP.

In conclusion, in this nationwide population-based longitudinal cohort study, we found that diabetes status is a predictor of glaucoma development in postmenopausal women. Our study suggests that diabetes status can be utilized to select higher-risk groups for glaucoma screening.

## Data Availability

Data are available from the Korea National Health Insurance Sharing Service Institutional Data Access Committee (https://nhiss.nhis.or.kr/bd/ay/bdaya001iv.do) for researchers who meet the access criteria.
